# Recurrent laryngeal nerve monitoring by flexible laryngoscopy during thyroid radiofrequency ablation in the awake patient

**DOI:** 10.3389/fendo.2024.1403087

**Published:** 2024-09-16

**Authors:** Marsida Teliti, Antonio Occhini, Rodolfo Fonte, Laura Croce, Benedetto Calì, Federica Antonella Ripepi, Andrea Carbone, Mario Rotondi, Spyridon Chytiris

**Affiliations:** ^1^ Department of Internal Medicine and Therapeutics, University of Pavia, Pavia, Italy; ^2^ Unit of Endocrinology and Metabolism, Laboratory for Endocrine Disruptors, Istituti Clinici Scientifici Maugeri IRCCS, Pavia, Italy; ^3^ Department of General and Minimally Invasive Surgery, Istituti Clinici Scientifici Maugeri IRCCS, Pavia, Italy; ^4^ Unit of Diabetology and Endocrinology, Medical-Oncologic Department, ASST Lodi, Lodi, Italy

**Keywords:** flexible laryngoscope, radiofrequency ablation, recurrent laryngeal nerve, bilateral, thyroid nodule

## Abstract

**Objective:**

Although radiofrequency ablation (RFA) is a safe and effective non-surgical treatment for benign thyroid nodules, injury to the recurrent laryngeal nerve (RLN), is a potential and feared complication. Intermittent voice checks have been proposed to monitor vocal cord (VC) function during RFA, but such assessment is highly subjective and effort-dependent.

**Methods:**

We are here reporting the methodological use of flexible laryngoscopy (FL) for VC monitoring during bilateral thyroid RFA treatment. The patient, a 35-year-old woman, was referred to the Endocrinology Unit for subclinical hyperthyroidism due to bilateral autonomously functioning thyroid nodules.

**Results:**

At the end of the treatment of the first nodule, the FL performed by an otorhinolaryngologist specialist allowed evaluating VC function and ruling out possible paralysis before proceeding with the contralateral RFA treatment. The patient was awake during the entire procedure and well tolerated the laryngoscopic examination. The TSH serum evaluations performed one month and 9 months after the procedure assessed an euthyroid state (TSH 3.2 mIU/L and 2.8 mIU/L, respectively).

**Conclusion:**

During bilateral thyroid RFA the use of FL for VC monitoring treatment resulted in a safe, easy-to-perform, and effective strategy to minimize and anticipate RLN injury risk in the awake patient. The prevention of RLN damage is advisable in the case of single RFA treatment, while it should be strongly recommended when RFA is performed on bilateral nodules.

## Introduction

Radiofrequency ablation (RFA) is an attractive and safe non-surgical approach for the treatment of benign non-functioning nodules as well as autonomously functioning thyroid nodules (AFTN) (1, 2). In experienced hands, the procedure has a low rate of complications. Indeed, according to the most recent and comprehensive meta-analysis, in patients undergoing RFA for benign thyroid nodules the incidence of overall complications has been reported to be ~ 2.0%, being the rate of major complications ~1.3% (3, 4). Advertent injury of the recurrent laryngeal nerve (RLN), during the RFA procedure is one of the feared complications (5). However, the overall rate of transient or permanent voice change following RFA has been reported to be ~ 1.40% based on subjective voice examination (2). To minimize RLNs damage, it has been proposed to leave a cuff of unablated posterior thyroid tissue immediately adjacent to the tracheoesophageal groove, or so-called “danger triangle,” where the RLN is not visible but is expected to be present (1, 6). In addition, the slow injection of cold dextrose 5% for hydrodissection has proven to be an effective method for separating the target lesion from surrounding critical structures (4, 7). Lastly, the use of the ‘moving shot technique’ with a trans-isthmic approach further reduces the risk of this feared complication (1). However, despite these precautions, injury to the laryngeal nerves by their anatomical site remains a potential risk. Typically, patients present with hoarseness of voice during or immediately following the procedure. Indeed, intermittent voice checks have been proposed to monitor vocal cord (VC) function during RFA, but such assessment is highly subjective and effort-dependent (1). The observation of passive or active symmetrical VC movements during breathing or phonation by laryngeal ultrasonography is a further noninvasive and convenient method for assessing VC function (8). However, at present flexible laryngoscopy (FL) remains the gold standard exam for evaluating VC mobility and to anticipate potential RLN damage. At least theoretically, the prevention of RLN damage is advisable in the case of single RFA treatment, while it should be strongly recommended when RFA is to be performed on bilateral nodules. In this view, we here describe the methodological procedures of performing FL for vocal cord monitoring during RFA treatment of bilateral thyroid nodules.

## Case discussion

**Figure 1:**
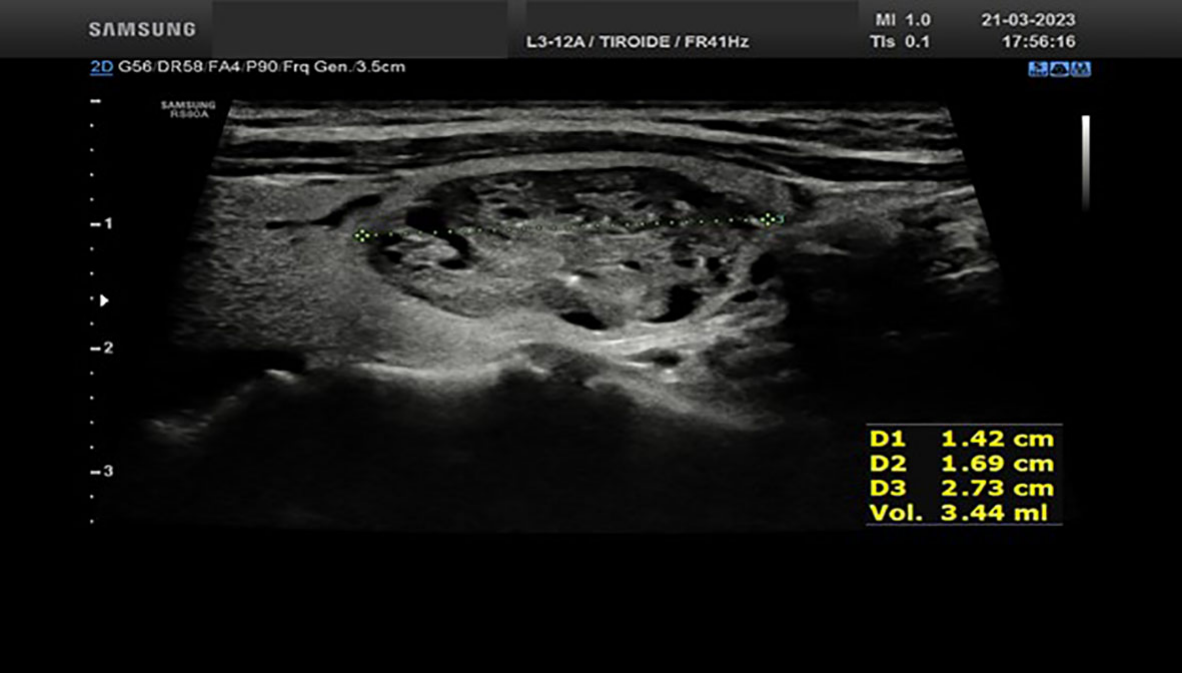
Ultrasound appearance of the thyroid right lobe nodule before RFA procedure.

**Figure 2:**
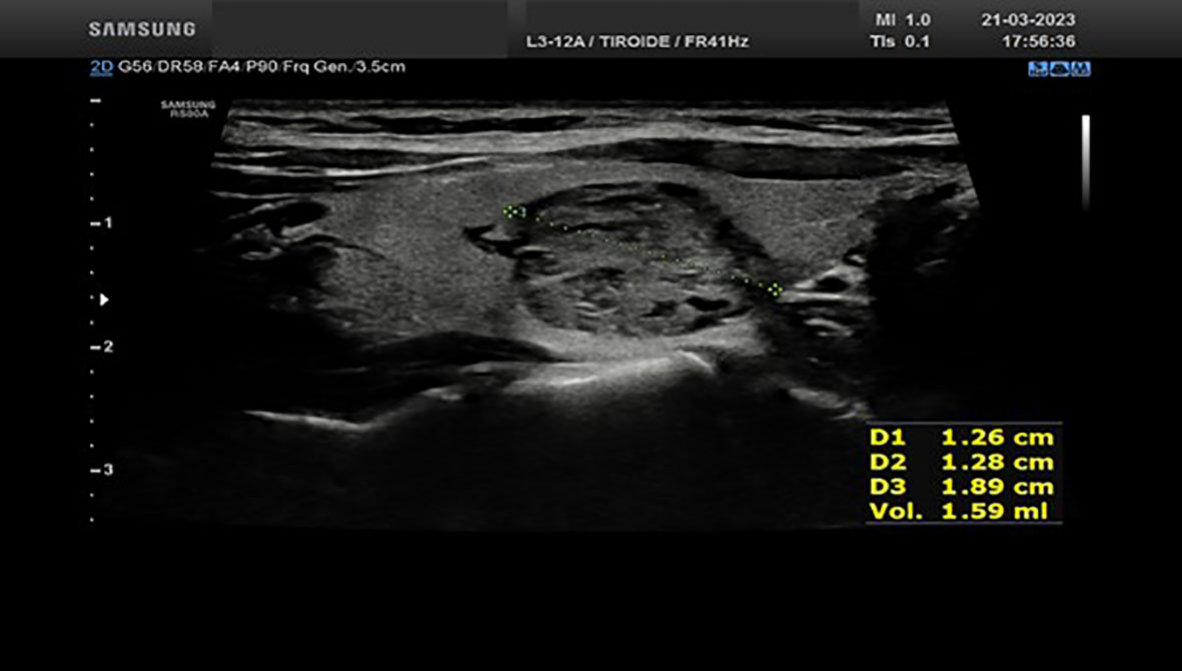
Ultrasound appearance of the thyroid left lobe nodule before RFA procedure.

We report the case of a 35-year-old woman who was referred to the Endocrinology Unit for subclinical hyperthyroidism diagnosed by a thyroid work-up performed for an assisted reproduction program. Throughout the last years, the patients experienced a progressive decrease in serum TSH (from 0.74 mIU/ml to 0.19 mIU/ml at the last evaluation performed on March 2023). The thyroid ultrasound, performed by the same endocrinologist (S.C) who performed the RFA procedures, showed a normal sized gland in the presence of two nodules. The first nodule was located in the basal area of the right thyroid lobe, being hypoechoic, partially cystic, and internally vascularized, with diameters of 14.2x16.9x27.3 mm and an estimated volume of 3.44 ml (**Figure 1**); the second one, with the same ultrasound features, was located in the middle third of the left thyroid lobe, with diameters of 12.6x12.8x18.9 mm and an estimated volume of 1.59 ml (**Figure 2**). Both nodules corresponded to areas of increased radioisotope uptakes at thyroid scintiscan (without inhibition of the surrounding parenchyma). The patient was offered the following therapeutic options: 1) thyroid surgery (i.e. total thyroidectomy); 2) radioiodine ablation therapy; 3) ablation of the thyroid nodules by radiofrequency. The advantages and disadvantages of each therapeutic option were discussed with the patient. Owing to the fact that the patient was planning pregnancy and was starting a medically assisted reproduction program, radiofrequency ablation of both nodules appeared as the most suitable option.

## Methods

RFA procedure was carried out on April 2023 in a sterile setting. Local anesthesia with Lidocaine 2% was administered at the skin puncture site and the perithyroidal space. No hydrodissection or anesthetic infusion was made in the peri- or under-capsular layer. The operator (S.C) was an endocrinologist with over 20 years of experience in thyroid imaging, fine-needle aspirations, core-needle biopsies, and percutaneous ethanol therapy who performs RFA treatment since 2018. During the procedure, the patient remained in a supine position with mild neck extension. The procedure was performed using an 18-gauge internally cooled electrode, 7 cm length with a 10 mm active tip. The US-guided moving-shot technique with a trans-isthmic approach was applied. At first, the right lobe nodule was treated (8.4 kJ were delivered in 9 min and 56 sec for a total of 8 ablations). The patient was advised to report pain, and voice testing was performed at regular intervals. When complete right nodule ablation was achieved, FL was performed by an otorhinolaryngologist. Briefly, FL was performed using a 30 cm long flexible Rhino-Laryngo-Fiberscope with a diameter of 3.5 mm. The patient was seated during the procedure. Before beginning, the patient was instructed to close her mouth and breathe gently through her nose. The tip of the endoscope was then advanced along the floor of the nose. The fiberscope was then maneuvered over the soft palate, allowing visualization of the vocal cords. This enabled the assessment of vocal cord mobility, to assess the normal motility of the VC. VC function was evaluated between the two procedures. Afterwards, treatment of the nodule at the left lobe proceeded (9.3 kJ were delivered in 10 min and 50 sec for a total of 5 ablations). A few hours after an US of the neck was performed: the right lobe nodule measures were 16.3x20x25.7 mm vs 14.2x16.9x27.3 mm (estimated volume 4.39 ml vs 3.44 ml); the left lobe nodule measures were 15.6x14.9x16.4 mm vs 12.6x12.8x18.9 mm (estimated volume 1.99 ml vs 1.59 ml). Minimal perithyroid inflammatory tissue was present with no visible hematomas. The absence of dysphonia after the end of the RFA procedure did not prompt us to repeat FL.

## Results

FL performed at the end of the right thyroid nodule’s RFA treatment recorded normal right VC function. The patient was awake in the surgery room. Symmetric spontaneous and volitional VC movements, including swallowing, were documented before proceeding with the treatment of the contralateral nodule. The patient well tolerated the procedure.

### Ultrasound and biochemical follow-up

**Figure 3:**
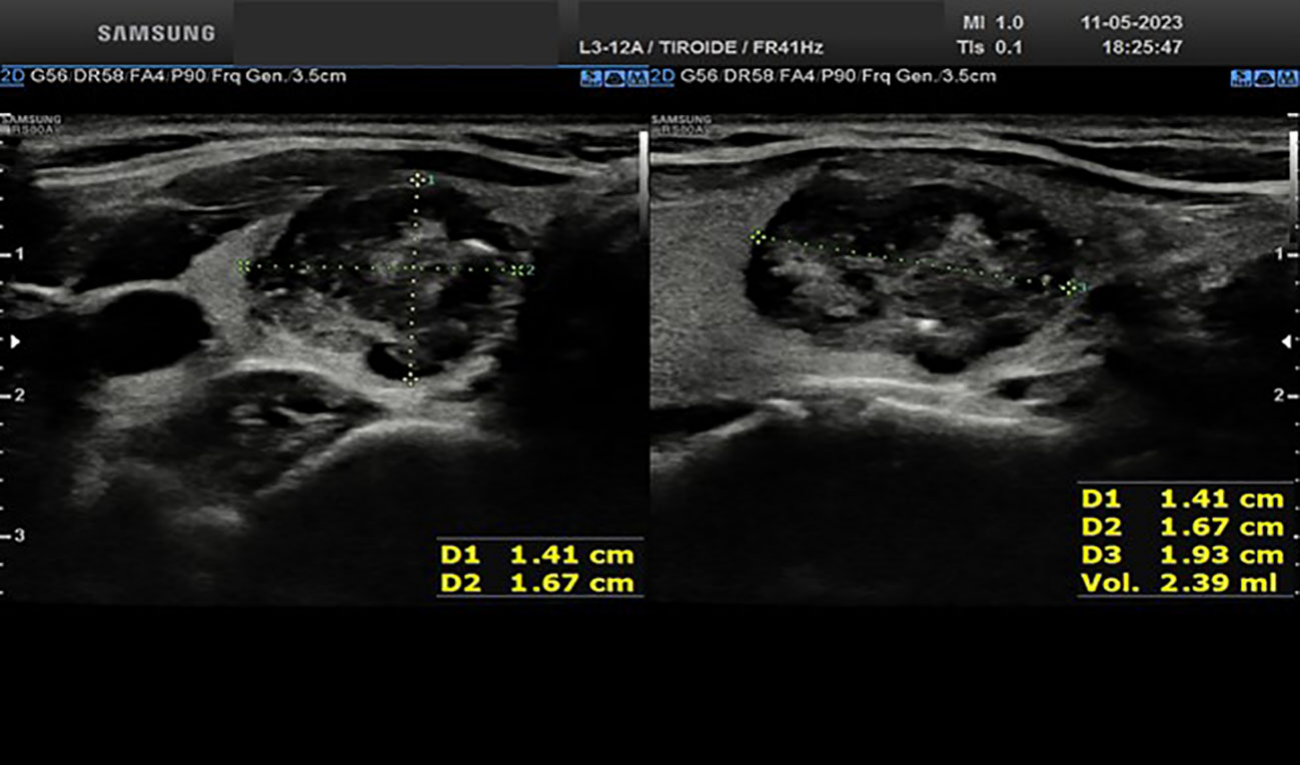
Ultrasound appearance of the thyroid right lobe nodule one month after RFA procedure.

**Figure 4:**
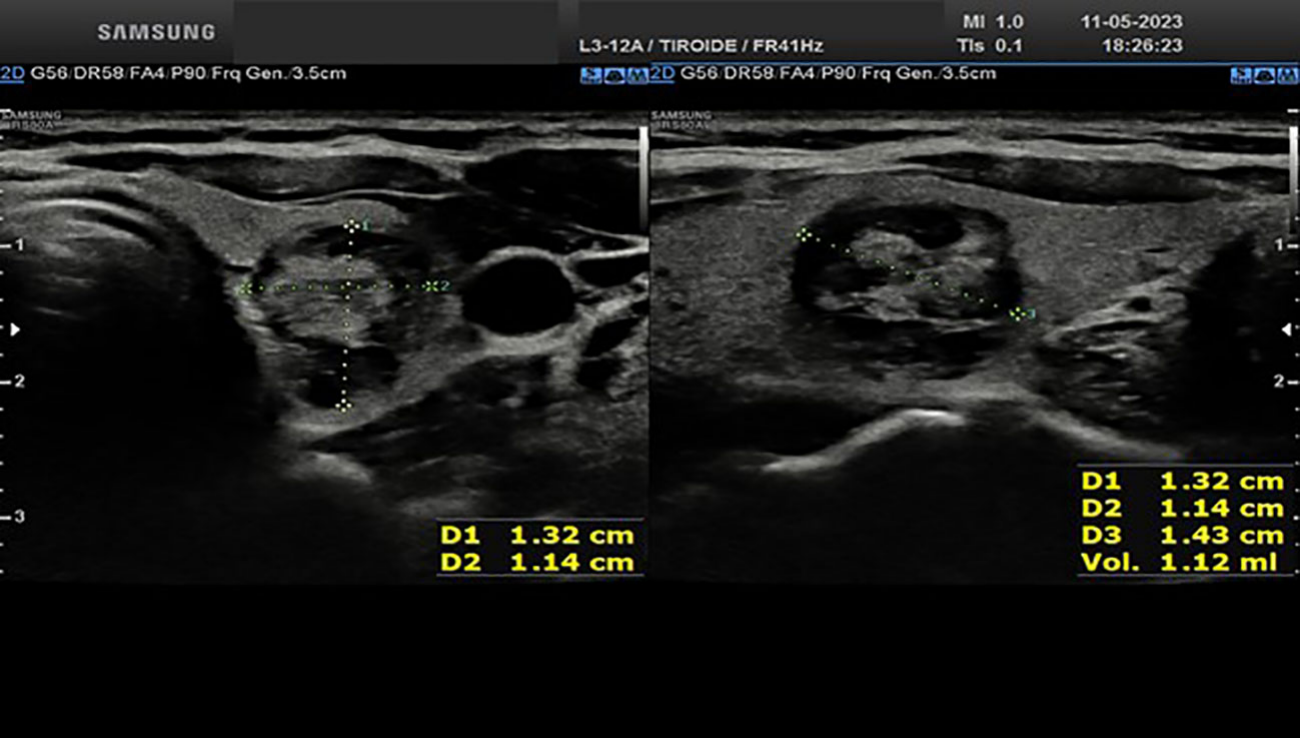
Ultrasound appearance of the thyroid left lobe nodule one month after RFA procedure.

Approximately one month after the procedure, the right basal nodule measures 14.1x16.7x19.3 mm (estimated vol 2.39 ml vs 3.44 ml, **Figure 3**); the medium left nodule measures 13.2x11.4x14.3 mm (estimated vol 1.12 ml vs 1.59 ml, **Figure 4**). Both nodules appear completely treated, occupied by a hypoechoic area, a result of the treatment. One month after the procedure, the thyroid function was restored (TSH 3.2 mIU/L). At the last serum TSH assessment on January 2024, euthyroidism was confirmed (TSH 2.8 mIU/L).

## Conclusion

We report the use of FL for VC monitoring during RFA treatment of bilateral functioning thyroid nodules in the awake patient. At the end of the treatment of the first nodule, the laryngoscopic examination allowed evaluating VC function and ruling out possible paralysis before proceeding with the contralateral procedure.

Recently, the use of FL for VC monitoring during RFA has been documented by Valcavi et al. for a patient under general sedation with a large thyroid nodule (9). FL was performed throughout the thyroid RFA to support the operator during the procedure. In the absence of documented thermal injury, the operator was comfortable in proceeding with extensive ablation of the nodule. The Authors propose that this approach should be recommended for large thyroid nodules to reduce the risk of undetected injury during the procedure. Of note, Fung et al. (8), first reported the use of intra-operative laryngeal ultrasonography (iLUSG) during thyroid RFA. Compared to FL, transcutaneous laryngeal ultrasound can offer some advantages including the fact that it is a non-invasive and easy-to-learn method. More importantly, iLUSG would not require the involvement of an otolaryngologist, being easily performed by physicians skilled in neck ultrasound. However, some limitations to the use of iLUSG should be acknowledged. Indeed, previous evidence showed that the presence of calcified laryngeal cartilage, which is mostly found in older male patients, could prevent accurate VC function assessment (10, 11). Nevertheless, the role of ultrasound in this context is highly promising and certainly warrants further investigation (12). The issue of potential VC damage following RFA is particularly relevant when bilateral nodules are treated, as VC function can be normal during treatment, but it may occur post treatment due to the procedure-induced edema.

Some peculiarities of the here reported case deserve to be highlighted: i) the patient was awake; ii) early detection of RFA-induced VC paralysis would have prevented potential bilateral injury, as undetected injury during RFA may result in unilateral or, even worse, bilateral RLN damage. Taken together it could be suggested that independently of nodule size, FL should be performed when treating bilateral nodules. Indeed, the use of a trans-isthmic approach even by an experienced operator may not be sufficient to prevent RLN injury. Thus, an additional measure of safety could be represented by a direct evaluation of VC functional integrity before proceeding with the treatment of the contralateral nodule. If RLN injury is detected, the procedure can be stopped immediately to limit further neural damage, and timely remedies such as intra-operative injection of cold 5% dextrose can be performed (13).

In conclusion, we are reporting the first case of the use of FL for VC monitoring during bilateral thyroid RFA treatment as a safe, easy to perform, and effective strategy to minimize RLN injury risk in the awake patient.

## Data Availability

The original contributions presented in the study are included in the article/supplementary material, further inquiries can be directed to the corresponding author.
